# Delusional Jealousy (Othello Syndrome) in 67 Patients with Parkinson’s Disease

**DOI:** 10.3389/fneur.2018.00129

**Published:** 2018-03-07

**Authors:** Hiroshi Kataoka, Kazuma Sugie

**Affiliations:** ^1^Department of Neurology, Nara Medical University, Kashihara, Japan

**Keywords:** Parkinson, delusional jealously, Othello syndrome, psychosis, dopamine agonist, delusion

## Abstract

Othello syndrome (OS) is a type of paranoid delusional jealousy, characterized by the false absolute certainty of the infidelity of a partner. Because OS has infrequently occurred in patients with Parkinson’s disease (PD), the characteristics of OS in PD remain unclear. We reviewed the clinical characteristics of this syndrome in PD. We reviewed 67 patients who had PD with OS. OS was more common in men (45 patients) than in women (22 patients), and it frequently occurred in middle-aged patients. Until the onset of OS, the duration of PD (range, 2–19.8 years) and the duration of treatment with PD medications (range, 2 months to 18.5 years) varied. At the onset of OS, cognition was preserved in most patients. 42 of 47 patients had other psychiatric disorders in addition to OS, and 5 patients had isolated OS. Persecutory or other paranoid delusions developed in 34 patients with OS. OS was associated with PD medication in 25 of 26 patients, especially in patients, used the dopamine agonists. The dose of the PD medication associated with OS was decreased or these drugs were withdrawn to facilitate the treatment of OS. In most patients, OS disappeared or the severity of OS was reduced. OS is infrequent in patients with PD, but is likely to be easily detected because OS is commonly accompanied by persistent paranoid and sexual delusions. When clinicians encounter such patients, the withdrawal or reduction of dopamine agonists should be attempted, and if necessary, additional treatment with clozapine is recommended.

## Introduction

Psychosis in Parkinson’s disease (PD) can severely interfere with the quality of life and care of patients. Psychosis is the most frequent cause of admission to a nursing home among patients with PD (1). More than 20% of all patients with PD are affected by psychosis (2). Typically, psychosis occurs in the late stage of PD and psychotic disorders may be present from the early stages (3). The most common symptoms in PD-related psychosis are visual hallucinations. Othello syndrome (OS) is a type of paranoid delusional jealousy, characterized by the false absolute certainty of the infidelity of a partner, leading to preoccupation with a partner’s sexual unfaithfulness based on unfounded evidence (4). OS is accompanied by irrational thoughts and emotions associated with unacceptable or extreme behavior. Because OS has infrequently occurred in patients with PD, the characteristics of OS in PD remain unclear. We focused on OS in PD and reviewed the clinical characteristics of this syndrome.

## Materials and Methods

PubMed was searched for relevant publications. The following keywords were used: delusional jealousy, OS, psychosis, delusions, jealousy, and Parkinson. The contents of publications in English were reviewed and appropriate articles were included. The references from the reviewed articles were examined for any missed articles. We newly found five case reports, four case series (5–11), and three studies (12–14) in addition to our previous case series (15). We added a new case treated in our institution. We reviewed 67 patients who had PD with OS as shown in Tables 1–3.

**Table 1:** Clinical characteristics of OS in 67 patients with PD (part 1).

	Sex	Age at PD onset	Age at OS onset	Disease duration (years)	Duration of PD therapy (years)	Hoehn–Yahr stage	UPDRS part 3	MMSE	Depression
Patient 1 [Ref. (5)]	M	45	51	7	5	2.5	14	30, very mild cognitive decline^a^	NA
Patient 2 [Ref. (5)]	M	48	51	4	3	2.5	16	30, no cognitive decline^a^	NA
Patient 3 [Ref. (5)]	M	49	55	7	5	3	18	28, very mild cognitive decline^a^	NA
Patient 4 [Ref. (5)]	M	54	56	3	1	2.5	16	30, very mild cognitive decline^a^	NA
Patient 5 [Ref. (5)]	M	45	52	8	6	3	18	27, very mild cognitive decline^a^	NA
Patient 6 [Ref. (5)]	M	44	49	6	5	2.5	19	28, very mild cognitive decline^a^	NA
Patient 7 [Ref. (6)]	M	51	44	7	5	2.5	19	29, very mild cognitive decline^a^	NA
Patient 8 [Ref. (7)]	M	68	74	6	4.5	Moderate parkinsonism	NA	NA	−
Patient 9 [Ref. (8)]	F	47	49	2	2 months	NA	NA	28	−
Patient 10 [Ref. (9)]	F	49	53	4	1	NA	NA	No cognitive decline	−
Patient 11 [Ref. (10)]	F	48	58	10	8	Worsened	NA	Poor recall	+
Patient 12 [Ref. (11)]	M	49	58	9	NA	NA	NA	Normal^b^	NA
Patient 13 [Ref. (11)]	M	39	42	3	NA	NA	NA	Mild dementia^b^	NA
Patient 14 [Ref. (11)]	F	53	64	11	NA	NA	NA	Very mild dementia^b^	NA
Patient 15 [Ref. (11)]	M	43	49	6	NA	NA	NA	Normal^b^	NA
Patient 16 [Ref. (11)]	M	50	56	6	NA	NA	NA	Very mild dementia^b^	NA
Patient 17 [Ref. (11)]	F	43	56	13	NA	NA	NA	Mild dementia^b^	NA
Patient 18 [Ref. (11)]	M	49	51	2	NA	NA	NA	Normal^b^	NA
Patient 19 [Ref. (15)]	F	42	59	17	9.75	3	23	30	−
Patient 20 [Ref. (15)]	F	59	79	19.8	18.5	4	17	17	−
Patient 21 [Ref. (15)]	M	58	65	7.5	6.4	3	13	28	−
Patient 22	F	66	73	7	6	3	43	16	−
Patient 23 [Ref. (12)] Patient 24 [Ref. (12)] Patient 25 [Ref. (12)] Patient 26 [Ref. (12)] Patient 27 [Ref. (12)]	2 F, 3 M	46.8 ± 8.87	56.4 ± 8.76	10.8 ± 9.41	6.25 ± 4.72^a^	NA	NA	None had cognitive decline	NA
15 patients [Ref. (13)]	6 F, 9 M	51.3 ± 7.5	61.2 ± 6.1	9.9 ± 5.2	NA	2.0 ± 0.6	18.0 ± 3.2	28.9 ± 1.3	10.6 ± 6.7 (Beck depression inventory)
5 patients [Ref. (13)]	5 M	60.4 ± 10.6	71.4 ± 7.0	11.0 ± 7.2	NA	2.4 ± 1.1	20.2 ± 7.1	20.2 ± 1.7	NA
20 patients [Ref. (14)]	6 F, 14 M	53.5 ± 9.0	63.7 ± 7.6	NA	NA	2.1 ± 0.8	NA	NA	NA

*PD, Parkinson’s disease; OS, Othello syndrome; UPDRS, unified Parkinson’s disease rating scale; MMSE, min-mental state examination;, NA,: not available*.

*^a^Global deterioration scale*.

*^b^Clinical dementia rating scale (CDR-SB)*.

**Table 2:** Clinical characteristics of OS in 67 patients with Parkinson’s disease (part 2).

	Family history of psychiatric disorders	Psychiatry history	Hs	Other associated psychiatric disorders	Influence on daily activity	MRI-CT cerebral atrophy
Patient 1 [Ref. (5)]	−	NA	−	−	NA	−
Patient 2 [Ref. (5)]	−	NA	−	−	NA	−
Patient 3 [Ref. (5)]	−	NA	−	−	NA	Mild
Patient 4 [Ref. (5)]	+	NA	−	−	NA	−
Patient 5 [Ref. (5)]	+	NA	+	Incest	NA	−
Patient 6 [Ref. (5)]	−	NA	+	Paraphilic behavior, frotteurism	NA	−
Patient 7 [Ref. (6)]	NA	−	−	Persecutory delusions, hypersexuality	+	NA
Patient 8 [Ref. (7)]	NA	−	−	Delusions	NA	NA
Patient 9 [Ref. (8)]	NA	NA	−	Persecutory paranoid ideation	+	NA
Patient 10 [Ref. (9)]	−	−	−	Delusions	+	−
Patient 11 [Ref. (10)]	NA	−	+	Delusions	+	NA
Patient 12 [Ref. (11)]	NA	−	NA	Hypersexuality	NA	NA
Patient 13 [Ref. (11)]	NA	−	NA	Hypersexuality, pathological gambling	NA	−
Patient 14 [Ref. (11)]	NA	Anxiety	NA	Pathological shopping	NA	−
Patient 15 [Ref. (11)]	NA	Depression	NA	Hypersexuality	NA	−
Patient 16 [Ref. (11)]	NA	−	NA	Hypersexuality, pathological gambling	NA	NA
Patient 17 [Ref. (11)]	NA	Anxiety	NA	Hypersexuality	NA	−
Patient 18 [Ref. (11)]	NA	Anxiety	NA	Hypersexuality, pathological shopping	NA	−
Patient 19 [Ref. (15)]	−	−	+	Persecutory delusions	+	−
Patient 20 [Ref. (15)]	−	−	+	Persecutory delusions	+	Both occipitals^a^
Patient 21 [Ref. (15)]	−	−	+	Persecutory delusions	+	Both occipitals^a^
Patient 22	−	−	+	Persecutory delusions	+	Both occipitals and frontals^a^
Patient 23 [Ref. (12)]	−	−	+	−	+	NA
Patient 24 [Ref. (12)]	−	−	−	Persecutory delusions	+	NA
Patient 25 [Ref. (12)]	−	−	−	Persecutory delusions	+	NA
Patient 26 [Ref. (12)]	−	−	+	Persecutory delusions	+	NA
Patient 27 [Ref. (12)]	−	−	−	Persecutory delusions	+	NA
15 patients [Ref. (13)]	7 Patients positive	Bipolar disorder (1)	Two patients	Delusions in all, pathological gambling (2), hypersexuality (1)	NA	NA
5 patients [Ref. (13)]	1 Patients positive	Anxiety disorder (1)	Four patients	Delusions in all, hypersexuality (3), panic (1)	NA	NA
20 patients [Ref. (14)]	NA	NA	NA	NA	NA	NA

*Hs, hallucinations; NA, not available*.

*^a^Decreased accumulation on single-photon emission computed tomography*.

**Table 3:** Association of anti-parkinsonian medications with OS in 67 patients with PD.

	PD medication associated with OS	Treatment of OS	Atypical neuroleptics used	Follow-up periods after treatment of OS	Outcome of OS
Patient 1 [Ref. (5)]	RP	RP reduction	Clozapine	NA	Reduced
Patient 2 [Ref. (5)]	PRX	PRX reduction	Clozapine	NA	Disappeared
Patient 3 [Ref. (5)]	Levodopa, pergolide, AMT	Pergolide reduction, withdrawal of AMT	Clozapine	NA	Reduced
Patient 4 [Ref. (5)]	PRX	Withdrawal of PRX	Quetiapine	NA	Reduced
Patient 5 [Ref. (5)]	Levodopa	Levodopa reduction	Clozapine	NA	Reduced
Patient 6 [Ref. (5)]	Pergolide	Withdrawal of pergolide	Quetiapine	NA	Reduced
Patient 7 [Ref. (6)]	Pelgolide	Pelgolide reduction	Quetiapine	NA	Disappeared
Patient 8 [Ref. (7)]	AMT	Withdrawal of AMT	−	4 years	Disappeared
Patient 9 [Ref. (8)]	PRX (2 months before OS)	Switch from PRX to levodopa	Quetiapine	3 months	Disappeared
Patient 10 [Ref. (9)]	PRX (2 months before OS)	Withdrawal of PRX	Quetiapine	3 months	Reduced
Patient 11 [Ref. (10)]	NA	Withdrawal of agonist, ECT, rivastigmine^a^	Quetiapine	NA	Reduced^a^
Patient 12 [Ref. (11)]	PRX	Withdrawal of PRX	−	NA	Disappeared or reduced
Patient 13 [Ref. (11)]	PRX	Withdrawal of PRX	−	NA	
Patient 14 [Ref. (11)]	PRX	Withdrawal of PRX	−	NA	
Patient 15 [Ref. (11)]	RP	Withdrawal of RP	−	NA	
Patient 16 [Ref. (11)]	PRX	Withdrawal of PRX	−	NA	
Patient 17 [Ref. (11)]	PRX	Withdrawal of PRX	−	NA	
Patient 18 [Ref. (11)]	RP	Withdrawal of RP	−	NA	
Patient 19 [Ref. (15)]	Levodopa (100 mg/day) increased (4, 5 months before OS)	Withdrawal of TRX, AMT reduction; PER reduction	Quetiapine	6.5 years	Disappeared
Patient 20 [Ref. (15)]	RP (8 months before OS), selegiline (11 months before OS)	Withdrawal of resuspension	Quetiapine	5 years	Disappeared
Patient 21 [Ref. (15)]	Not detectable	Withdrawal of ropinirole	Quetiapine	4.5 years	Disappeared
Patient 22	Rotigotine (1 weeks before OS)	Withdrawal of rotigotine	Quetiapine	2.5 years	Disappeared
Patient 23 [Ref. (12)]	Levodopa, apomorphine, PRX	Reduction of dopamine agonists	Clozapine, quetiapine	NA	Reduced
Patient 24 [Ref. (12)]	Levodopa, apomorphine, entacapone	Reduction of apomorphine	Quetiapine	NA	Not improved
Patient 25 [Ref. (12)]	PRX	PRX reduction	−	NA	Disappeared
Patient 26 [Ref. (12)]	RP	RP reduction	Clozapine	NA	Not improved
Patient 27 [Ref. (12)]	RP	Withdrawal of RP	−	NA	Disappeared
15 patients [Ref. (13)]	NA	Agonist withdrawal (2) or reduced (13)	Clozapine (7), quetiapine (4), aripiprazole (1)	NA	Disappeared (1, aripiprazole), partial resolusion (14)
5 patients [Ref. (13)]	NA	Agonist withdrawal or reduced (4)	Clozapine (2), quetiapine (3)	NA	Disappeared (2), partial resolusion (1)
20 patients [Ref. (14)]	NA	NA	NA	NA	NA

*PD, Parkinson’s disease; OS, Othello syndrome; PRX, pramipexole; RP, ropinirole; AMT, amantadine; TRX, trihexyphenidyl; NA, not available*.

*^a^PD medication modification or quetiapine failure*.

## Results

### Clinical Characteristics of 67 Patients

Othello syndrome was more common in men (45 patients) than in women (22 patients) and frequently occurred in middle-aged patients. 3 elderly patients older than 70 years also had OS. 6 patients had a history of psychiatric disorders and 10 patients had a family history of psychiatric disorders. Until the onset of OS, the duration of PD (range, 2–19.8 years) and the duration of treatment with PD medications (range, 2 months to 18.5 years) varied, and OS developed in two patients (patient 9 and 10) after receiving pramipexole for 2 months. At the onset of OS, the severity of PD (median UPDRS motor score, 18) was relatively mild and patients with an early stage of PD (Hoehn–Yahr 2nd or 3rd stage) frequently presented with OS. One patient (patient 20) had late stage PD with a Hoehn–Yahr score higher than 4. At the onset of OS, cognition was preserved in most patients, but some patients had mild-to-moderate cognitive impairment. Beck Depression Inventory (no depression ≤10) was mildly increased in 15 patients (13). In addition to OS, 42 of 47 patients had other psychiatric disorders as follows: hallucinations in 15, depression in 1 (patient 11), or impulse-control disorders (16) in 14 patients. Eleven patients had hypersexuality, pathological gambling was present in four patients, and pathological shopping was evident in two patients. Persecutory or other paranoid delusions developed in 34 patients with OS. Five patients had isolated OS. Most cases of OS adversely affected the daily lives of the patients.

### Associated Parkinsonian Medications

Othello syndrome was associated with PD medications in 25 of 26 patients. In particular, 22 patients used the following dopamine agonists before the onset of OS: pramipexole in 11 patients, ropinirole in 6 patients, pergolide in 3 patients, apomorphine in 2 patients, and rotigotine in 1 patient. Other PD medications associated with OS were levodopa in five patients, amantadine in two patients, selegiline in one patient, and entacapone in one patient. OS was not associated with PD medication in one patient.

### Treatments and Outcomes

In 47 of the 47 patients, the dose of the PD medication associated with OS was decreased, or these drugs were withdrawn to facilitate the treatment of OS. In one patient, pramipexole was switched to levodopa. 35 of 47 patients received atypical neuroleptics, such as quetiapine (19 patients) or clozapine (15 patients). Electroconvulsive therapy was effective in one patient. In 43 patients, OS disappeared or the severity of OS was reduced. Seven patients remained free of OS.

## Discussion

### Clinical Aspects

Othello syndrome is an infrequent form of psychosis in PD. However, the interval between the time that OS symptoms are first recognized by the patient and OS is diagnosed by a medical professional was reported to be 4 months (12). If the duration of OS is short or OS has occurred several times, it may be missed. However, many cases of OS are prolonged or associated with exaggerated behavioral changes of the patients, their spouses, or both. In such cases, OS could be easily recognized because the spouses of the patients consulted physicians to confirm the diagnosis. Also, among the many forms of paranoid delusions, OS is known to be most associated with the belief of spousal infidelity (5). The estimated incidence of OS in PD was reported to be 5.2% among 116 patients with PD (17), 1.1% among 563 non-demented patients with PD (5), or 2.48% among 805 patients with PD (14). OS generally occurs in middle-aged patients (5, 14) and younger age was reported to be a risk factor for OS (14). Our review also showed that middle-aged patients frequently presented with OS. OS developed not only in patients with the early onset of the parkinsonian symptoms, but also in patients with advanced PD. Most cases of OS occurred in non-demented patients with PD.

On the basis of the neuropsychiatric assessments, OS is classified into the following subgroups: demented patients, cognitively preserved patients with a relatively early onset of PD, and cognitively preserved patients in whom OS appeared after dopaminergic medication of variable duration (13). Another study mentioned four subtypes of psychosis in PD (18). Psychotic symptoms occur (1) secondary to environmental factors other than PD, such as medical illness, hospitalization, surgery, or non-dopaminergic medication, but this was not seen in our review of OS. (2) Isolated OS or dopaminergic-induced OS, and (3) cholinergic disease-related psychosis or psychosis related to cognitive decline can occur. In our review, cognition was preserved in most patients as reported previously (13), and one cross-sectional study showed that the estimated rate of the dementia among PD patients with OS is similar to that of PD patients without OS (14). The two subtypes are seen in patients with OS. (4) Psychosis related to affective disorders or serotonergic psychosis, such as anxiety, depression, or both was evident in some patients with OS in our review. OS usually did not present as an isolated condition, but was often accompanied by hallucinations appearing independently of OS. Delusions with concomitant hallucinations can be found in patients with PD, and hallucinations without retained insight may progress into more complex and severe psychotic symptoms, including delusions (19). Aberrant sexual behaviors or impulse-control behaviors (16) are more common in patients who use dopamine agonists.

### Associated Parkinsonian Medications

All dopaminergic medications carry the risk of eliciting OS as well as psychotic symptoms. A poor correlation between specific dopaminergic drugs and the onset of OS was reported, but the sample was small (5). In our review, dopamine agonists were related to OS in most patients, consistent with the results of previous studies (5, 8, 12, 14). Also, the younger patients with preserved cognition who had OS had received treatment with a dopamine agonist, suggesting that OS is closely related to dopamine agonists. Evidence of a relation between the use of dopamine agonists and age in patients with OS is lacking, but patients who were younger and more frequently received dopamine agonists trended to have OS (14). The key dopamine receptor is most likely the D3 receptor, since the dopamine D3 receptor is involved in reward, craving, emotional, and cognitive processes (20). Pramipexole is a direct D3 agonist, and the use of pramipexole as an agonist is the most likely cause of OS, as reported previously (5, 11). Other agonists can also cause OS because ropinirole or pergolide also share D3 affinity. However, a distinct association between the duration or dose of dopamine agonists and the onset of OS remains uncertain. In one patient, levodopa monotherapy was associated with OS (5).

### Possible Pathophysiology

The pathophysiology of OS is unclear. Psychosis is probably caused by an increase in dopamine in the mesolimbic pathway (6, 21), which can potentially cause thought disruption. Increased dopaminergic sensitivity in mesolimbic and mesocortical pathways is involved in the generation of psychosis (22). A patient with PD who had hypersexuality showed the increased regional accumulation of dopamine in the right medial temporal regions on single-photon emission computed tomography, and after lowering the dose of pergolide, the increased accumulation disappeared (23). A higher density of Lewy pathology in the parahippocampus and amygdala (24) and α-synuclein in limbic regions (25) has also been described in PD. Moreover, D3 agonists induce a decrease in blood flow in the right frontal lobe (11). Dopaminergic excitation of the limbic region with reduced prefrontal stimulation might cause sensory input from multiple associated cortices to be misinterpreted, which can lead to psychosis, including OS (21). Other serotonergic and cholinergic transmitter systems are also involved (18). Confirmation of this hypothesis must await future studies.

### Treatments

Basically, the treatment of OS is similar to that of psychosis. Withdrawal or reduction of anti-parkinsonian medications is initially considered, and the best option is represented by the withdrawal of dopamine agonists or their replacement by equivalent doses of levodopa (13). In our review, OS disappeared in many patients. However, treatment was unsuccessful in some patients with OS, particularly those who had preserved cognitive function, owing to the rapid onset of a dopamine agonist withdrawal syndrome (26). Patients with persistent OS should be additionally given the antipsychotics clozapine or quetiapine if necessary. Other acetylcholinesterase inhibitors as well as atypical antipsychotics, such as risperidone or olanzapine have been tested. Acetylcholinesterase inhibitors can worsen PD symptoms, such as tremor, as compared with atypical antipsychotics. Clinicians should be aware that these treatment options can adversely affect the motor symptoms of PD.

As for the treatment of overall psychosis in PD, many open-label studies have attested to clozapine’s efficacy without impairment of motor function (27–29). Clozapine, in very low doses, was effective with dramatic reductions in psychosis, without significant changes in motor function in a randomized, double-blind placebo-controlled study (PSYCLOPS trial) (30). Only clozapine was given A level support for its use to treat psychosis in patients with PD by the Movement Disorders Society review committee on evidence-based medicine, 2011 version (31). In 2007 review by the American Academy of Neurology’s task force on the treatment of PD, clozapine and quetiapine were recommended for “consideration” only (32). There is no clinical trial evidence to support the efficacy of quetiapine. A single open-label study demonstrated that olanzapine was effective in patients with PD who had psychosis (33). However, olanzapine was reported to be ineffective for psychosis and worsened Parkinson motor features (34, 35). In April 2016, pimavanserin was approved by the Food and Drug Administration as a novel therapeutic option for psychosis in PD. Pimavanserin acts as an inverse agonist at serotonin 5-HT2A receptors and has negligible effects on other receptors, thereby avoiding worsening of motor symptoms (36).

In conclusion, OS is infrequent in patents with PD, but is likely to be easily detected because OS is commonly accompanied by persistent paranoid and sexual delusions. When clinicians encounter, such patients, the withdrawal or reduction of dopamine agonists should be attempted, and if necessary, additional treatment with clozapine is recommended.

## Author Contributions

HK was responsible for the overall study design and wrote the manuscript. HK contributed to the acquisition of data and to analysis and interpretation of the data. HK and KS contributed to drafting and critical revision of part of the submitted materials.

## Conflict of Interest Statement

The authors declare that the research was conducted in the absence of any commercial or financial relationships that could be construed as a potential conflict of interest.
